# Towards Inhaled Phage Therapy in Western Europe

**DOI:** 10.3390/v11030295

**Published:** 2019-03-23

**Authors:** Sandra-Maria Wienhold, Jasmin Lienau, Martin Witzenrath

**Affiliations:** 1 Division of Pulmonary Inflammation, Charité–Universitätsmedizin Berlin, Freie Universität Berlin, Humboldt-Universität zu Berlin, and Berlin Institute of Health, 10117 Berlin, Germany; sandra.wienhold@charite.de (S.-M.W.); jasmin.lienau@charite.de (J.L.); 2 Department of Infectious Diseases and Respiratory Medicine, Charité–Universitätsmedizin Berlin, Freie Universität Berlin, Humboldt-Universität zu Berlin, and Berlin Institute of Health, 10117 Berlin, Germany

**Keywords:** bacteriophage, phage therapy, multidrug-resistant bacteria, antimicrobial resistance

## Abstract

The emergence of multidrug-resistant bacteria constitutes a great challenge for modern medicine, recognized by leading medical experts and politicians worldwide. Rediscovery and implementation of bacteriophage therapy by Western medicine might be one solution to the problem of increasing antibiotic failure. In some Eastern European countries phage therapy is used for treating infectious diseases. However, while the European Medicines Agency (EMA) advised that the development of bacteriophage-based therapies should be expedited due to its significant potential, EMA emphasized that phages cannot be recommended for approval before efficacy and safety have been proven by appropriately designed preclinical and clinical trials. More evidence-based data is required, particularly in the areas of pharmacokinetics, repeat applications, immunological reactions to the application of phages as well as the interactions and effects on bacterial biofilms and organ-specific environments. In this brief review we summarize advantages and disadvantages of phage therapy and discuss challenges to the establishment of phage therapy as approved treatment for multidrug-resistant bacteria.

## 1. Introduction

Antimicrobial drug resistance (AMR) is a growing challenge worldwide. The emergence of new resistance mechanisms and their broad distribution through vertical and horizontal gene transfer is alarming. Consequently, multidrug-resistant (MDR) bacteria are spreading globally [1]. Due to the lack of a global tracking system, the full impact of infections with MDR bacteria is still unknown. A recent study estimated that approximately 33,000 people died in 2015 in the European Union as consequence of an infection with a resistant pathogen [2]. In the U.S. about 23,000 people die each year due to infections with resistant bacteria and far more people are infected [3]. Besides the medical aspect the socio-economic burden for health care systems is enormous [3]. Previously, the focus was mainly on gram-positive bacteria such as methicillin-resistant *Staphylococcus aureus* (MRSA) or vancomycin-resistant enterococci (VRE) but in recent years gram-negative bacteria resistant against 3 or 4 classes of antimicrobial drugs or even pan-resistant bacteria are rapidly gaining importance. In this respect, particularly noteworthy are *Pseudomonas aeruginosa* and *Acinetobacter baumannii* [4]. Many advanced therapies for cancer or autoimmune diseases, as well as transplantations are no longer effective when patients suffer from untreatable nosocomial infections [5]. Even commensal and opportunistic bacteria could then become problematic and jeopardize medical progress [6]. Moreover, novel antibiotics are rare as the pharmaceutical industry has minimized research and development programs in infectious diseases for different reasons [7]. Meanwhile, as AMR poses a major public health concern, this issue has been discussed at the highest political levels (from the United Nations and the WHO to local authorities). The “Leaders´ Declaration G7 Summit, 7–8 June 2015” (held in Elmau, Germany) stated: “We will foster the prudent use of antibiotics and will engage in stimulating basic research, research on epidemiology, infection prevention and control, and the development of new antibiotics, alternative therapies, vaccines and rapid point-of-care diagnostics” [8].

Already 100 years ago, a decade before the discovery of penicillin, bacteriophages (phages) were considered for clinical use [9]. However, driven by the easy use and broader antibacterial spectrum of antibiotics, phage therapy was seldom used during the last few decades, especially in Western Countries [10]. Only in some countries of the former Soviet Union, such as Georgia and Russia but also in Poland, have physicians continued to use phages and generated valuable practical experience [11]. The recent rediscovery and reintroduction of bacteriophage therapy in the Western World may possibly provide an attractive solution to the increasing failure of antibiotics. Since then, phages have been shown to be effective in treating bacterial infections in several experimental animal studies, as well as in case reports and clinical trials in humans [12]. In *Staphylococcus aureus* induced sepsis in mice for example, intraperitoneal (i.p.) application of phages 6 h after infection resulted in survival rates of 67%, whereas only 10% of control mice survived [13]. Systemic phage lysin application increased survival of mice with severe pneumonia due to *S. pneumoniae* from 0 to 100% [14]. Furthermore, it was also shown that inhaled application of the bacteriophage endolysin Cpl-1 is a safe and efficient therapy in severe pneumococcal pneumonia in mice [15]. So far, lysins seem to be more effective for treating gram-positive bacteria and are currently being tested in clinical trials [16]. Improvement of the enzymes’ penetrative abilities through the outer membranes is necessary for their efficient use in gram-negative bacteria [6,17].

In *P. aeruginosa* lung infection, intranasal application of phages, given 24 h prior or 2 h after infection, protected all mice from lethal infection [18]. In diabetic and nondiabetic mice with severe bacteraemia due to i.p. injection of MDR *P. aeruginosa*, a single i.p. injection of phages 20 min after bacterial injection increased survival [19]. The authors reported a survival rate of 90% in diabetic and 100% in nondiabetic mice even when treatment started 4 h after bacterial challenge. Treatment started 6 h after infection resulted in lower survival rates among diabetic mice. Further delay of treatment (12 h) also reduced the effectiveness of phage therapy in nondiabetic mice [19]. This suggests, phage therapy is effective in both immunocompetent and -incompetent mice. In the UK a clinical trial (double-blind placebo-controlled, randomized phase I/II) for treatment of chronic otitis media investigated the effect of a phage-cocktail of 6 phages against MDR *P. aeruginosa.* The study demonstrated physical improvement of the patients and distinctly lower *P. aeruginosa* counts compared to the placebo treated group after a single aural application. Notably, no side effects were reported [20]. These studies along with others indicate phage therapy could be a promising prospect for the treatment of MDR infectious diseases [21]. In laboratory animals, phages can generally be administered via different routes for example, i.p., subcutaneous (s.c.), intramuscular (i.m.), intravenous (i.v.), oral, inhaled or topical [22,23], with the success of phage therapy depending on both the application route and the target organ. After parenteral application, phages are quickly distributed in the systemic circulation [23,24]. McVay et al. [24] investigated different application routes for phage therapy in mice subjected to burn injury and subsequently infected with *P. aeruginosa.* Mice were treated i.m., s.c., i.p. or left untreated. In the untreated group only 6% of mice survived, whereas 28% and 22% of animals survived after i.m. or s.c. phage treatment, respectively. Intraperitoneal application yielded the highest effectiveness, resulting in 88% survival [24]. Oral application was shown to be effective in treating gastrointestinal infections [25,26], whereas topical application was successfully used to treat wound infections [27]. Nebulization of phages for inhaled application to treat lung infections has also been studied [28,29]. Huff et al. [30] reported that chickens, infected with *E. coli* into the thoracic air sac after pre-treatment with aerosolized phages showed significantly reduced mortality compared to untreated birds. However, Carmody et al. [31] demonstrated that intranasal inhalation of phages was less effective when compared to systemic application in a mouse model of lung infection caused by *Burkholderia cenocepacia*. Conversely, Semler et al. [32] observed that inhaled phage therapy was superior to i.p. injection in eliminating *Burkholderia cenocepacia* in murine lung infection.

Phages used for any medical application must be carefully selected and fully characterized [6]. Phages showing poor adsorption, replication and distribution should be excluded and exclusively obligate lytic phages should be applied [33]. Temperate phages may lead to the transfer of genes to the bacterial host, increasing its virulence by lysogenic conversion or transduction mechanisms or transferring virulence factors or antibiotic resistance genes from prophage genomes to the host bacteria [34,35]. The causative bacterial pathogen must be identified prior to phage selection, requiring fast and reliable pathogen detection and susceptibility screening [35]. Alternatively, bacteriophage cocktails including phages against the most common and typical pathogens in specific organs (e.g., “respiratory bacteria”) could be employed [6]. In any case, phage therapy specific infrastructure, such as local, rapidly accessible phage libraries need to be established [6,36].

Whereas studies on effective phage therapy have been reported and extensively reviewed, there are hardly any reports on phage therapy failures in recent years. Reports of failures mainly date back to the early use of phage therapy [37]. In 2001, Sulakvelidze et al. [38] published a detailed overview of phage therapy in Eastern European countries starting in the 1920s. The authors stated that failures occur mostly due to poor phage preparations, limited knowledge regarding phage mode of action and inconsistencies between phages and host strains. Additionally, most studies were lacking placebo controls leading to controversial results [38]. Miedzybrodzki et al. [39] published a summary of 153 patients treated with phage therapy to different infections between 2008 and 2010 at the Hirszfeld Institute of Immunology and Experimental Therapy in Wrocław. 39.9% of all patients showed a good response to phage therapy and in 18.3% pathogen eradication and/or recovery was reported [39]. Moreover, there is one clinical trial from Bangladesh using phage cocktails targeting *Escherichia coli* (*E. coli*) in children with bacterial diarrhoea reporting no advantage of phage therapy [40]. Two different phage cocktails were tested and orally applied [40]. Phage therapy did not cause any side effects but also did not improve the clinical outcome compared to the control group receiving standard oral rehydration. The authors reported several limitations of the trial, including probable insensitivity of pathogenic *E. coli* strains to the applied phages and the possibility of low stomach pH affecting phage transport, as no antacid was given to the patients [40].

In this review we summarize the advantages and disadvantages of phage usage in terms of medical application and discuss challenges to the establishment of phage therapy as an approved treatment for MDR bacteria.

## 2. Advantages and Disadvantages of Phage Therapy

### 2.1. Phage Therapy Provides Several Advantages Over Conventional Antimicrobial Drugs Regarding Medical Application, Some of Which Are Addressed in the Following

#### 2.1.1. Host Specificity and Potential to Spare Microbial Flora

Lytic bacteriophages are viruses that target and infect their specific host bacteria, replicate inside and destroy them [9]. Therefore, unlike indiscriminate antibiotics, bacteriophages are expected to spare the physiologically resident flora, thus avoiding the development of bacterial niches that typically result from antibiotic therapy and enable the settlement of antibiotic-resistant bacteria [41]. Moreover, an intact microbiome contributes to innate immunity vigilance [10,42,43]. Consequently, local gut immune response to bacterial challenge is dampened by preceding antimicrobial therapy, increasing susceptibility to intestinal colonization and infection with pathogenic microorganisms, including MDR bacteria [41]. Furthermore, disruption of the gut microbiome results in an impaired systemic immune response upon bacterial stimulation and ultimately insufficient bacterial elimination [44,45]. Thus, antibiotics [44] but not phage therapy may possibly compromise immunity through microbiome disruption, paving the way for subsequent MDR and non-MDR infections. However, this conjecture needs to be addressed in upcoming studies. Similarly interaction of resident phages (phageome) with commensals as well as function and dynamics of the phageome have not yet been completely unravelled [46].

#### 2.1.2. Bacterial Phage Resistance

Phage infection and lysis occur independently of mechanisms used by antibiotics to kill bacteria. Therefore, antibiotic resistance does not imply phage resistance [47]. Resistance to phages, however, occurs naturally and to varying degrees in all bacterial cultures and communities. Different mechanisms of resistance development have been described, including phage adsorption to bacterial cell receptors, phage particle assembly in the bacterial cell or cell lysis processes [48,49]. Notably, resources in the environment to isolate new phages are abundant [50]. Thus, isolation of new phages for almost all bacterial species can be achieved quickly for example, by sampling the environment and phage screening overnight (so called phagogram) [6] for subsequent GMP production. Consequently, development of pan-phage resistant bacteria is very unlikely [51]. Interestingly, it has been reported that some bacteria evolving phage resistance might over time regain sensitivity to antibiotics [52,53] or lose their virulence [54,55].

#### 2.1.3. Self-Replication, Self-Limitation and Anti-Biofilm Properties

Since bacteriophages can only target and infect their specific host bacteria, the lysis process is self-limiting [47]. Phages replicate as long as their host is accessible. Consequently, to initialize or continue the lytic cycle, phages need host bacterial cell contact, which is significantly impaired in the case of bacteria forming biofilms, remaining intracellularly or being less abundant. Phages encoding for depolymerases are able to degrade matrix exopolysaccharides of biofilms [56,57]. Consequently, phages and other antimicrobialsmight reach, infect and lyse bacteria inside the biofilm more easily [56,57], which is of particular interest in case of implants (e.g., vascular or joint devices) and airway infections [58,59,60]. Bedi et al. [61], showed a beneficial effect on the eradication of a biofilm formed by *Klebsiella pneumoniae* when antibiotic and phage were combined. Another study demonstrated that phage OMKO1 was able to reduce bacterial densities in a *P. aeruginosa in vitro* biofilm assay [62]. Moreover, this phage alone or in combination with an antibiotic was more effective in reducing the biofilm than antibiotics alone [62]. Nevertheless, the effectiveness depends on the phage and the host bacteria. Darch et al. [63] examined the ability of two phages to inhibit bacterial dissemination in a model of aggregate formation by *P. aeruginosa*. The two phages were able to kill *P. aeruginosa* and inhibit aggregate formation when applied simultaneously with the bacteria [63]. However, when applied after aggregate formation was already established, the authors did not observe complete elimination of aggregates, most likely due to exopolysaccharide production. Still phage application could prevent formation of new aggregates by proliferating bacteria [63]. Full elimination of a complex mature biofilm with one single phage seems unlikely but phage cocktails and combined therapy with antibiotics could be a potential strategy [56,57,64].

### 2.2. Some of the Disadvantages of Phage Therapy Are Addressed by the Following Aspects

#### 2.2.1. Activity against Intracellular Pathogens

Phages are unlikely to be able to actively enter eukaryotic cells. Therefore, phages are less effective against intracellular bacteria for example, *Mycobacterium tuberculosis*, as well as against intracellularly-surviving and persistent clones of extracellular bacteria, for example, *A. baumannii* [38].

#### 2.2.2. Liberation of Endotoxins

Although it seems unlikely that therapy with purified phages leads to relevant toxic side effects, major concerns encompass the potentially massive liberation of bacterial endotoxins after bacterial lysis. Similar observations have been made with the use of certain antibiotics [65], as well as immune reactions to bacterial components including endotoxin present in crude phage lysates [66]. Confrontation with large amounts of bacterial endotoxins could lead to clinical deterioration of septic patients [67,68]. However, Dufour et al [69] reported for two different *E. coli* phages fewer released endotoxins in vitro compared to β-lactams, while the phage-evoked endotoxin level was comparable to that evoked by amikacin [69].

#### 2.2.3. Potential Risk of Anaphylaxis

Phages are members of microbial communities and are present in the environment as well as on and in the human body [70,71]. Despite this, therapy with phages requires a higher titre compared to their naturally occurring numbers. Moreover, the use of high phage titres in patients bears the theoretical risk of inducing extreme immune responses like anaphylaxis [38]. Although theoretically possible, anaphylaxis due to phage therapy has never been reported and does not seem to be a major concern in phage therapy [11,47].

#### 2.2.4. Immune Response to Phages

Being composed of proteins and nucleic acids, phages in general are considered as innately non-toxic [47,72]. However, there is evidence for non-specific immunomodulatory characteristics of phages [73], as well as activation of phagocytosis and anti-inflammatory properties [74]. Roach et al. demonstrated that presence of neutrophils is necessary for phage therapy success against *P. aeruginosa* [75]. Moreover, a recent in vivo study revealed that an increased number of phages in the gut (applied via drinking water to mice) can aggravate colitis in a TLR9 and IFN-gamma dependent manner and that phages inside the gut could stimulate non-specific and phage-specific immunity [76].

It is also possible that the human immune system may recognize phages as foreign antigens and produce phage-neutralizing antibodies depending on the application route [77]. In order to minimize the risk of side-effects due to impurities, it is necessary in at least parenteral application routes to use highly purified phage preparations [78]. For a widespread use of human phage therapy according to Western European medical standards, more scientific evidence is needed. In particular further investigation is warranted in the areas of immunological reactions following single or repeated phage application, pharmacokinetics and -dynamics and interaction with bacterial biofilms and commensal flora.

## 3. Challenges in Clinical Use of Phage Therapy

### 3.1. Current State of Phage Therapy

Experience with human phage therapy dates back more than 100 years in Georgia, Russia and Poland. However, Jault et al [79] stated that these countries have not developed “evidence-based medical standards” so far and “if there are any, they are only available in Russian” [79]. Especially at the ELIAVA Institute of Bacteriophage in Tbilisi, significant effort has been put into the characterization and development of phage products. Phage cocktails are routinely used for treatment, including prescribed medicine and self-medication (over the counter products) [80,81]. In Poland, phage research is predominantly carried out at the Hirszfeld Institute of Immunology and Experimental Therapy in Wrocław [82]. The institute focuses on preparation of specific phage lysates for individual patients subsequent to identification of causative pathogens from patient’s samples. After Poland’s accession to the EU, the Phage Therapy Unit, an outpatient clinic working according to EU regulations, was established. In this unit phages are applied under terms of experimental treatment in accordance with the Declaration of Helsinki and Polish regulations [83,84]. Despite years of practical experience, numerous case reports [39,85,86,87] and data from clinical studies including investigations of immune response [20,88,89,90,91,92,93], the lack of peer-reviewed controlled clinical trials still renders an evidence-based evaluation of phage therapy by Western standards difficult [84]. Marketing authorization of phage therapy in Western Europe depends on several conditions, including production of phage preparations according to good manufacturing practice (GMP) conditions, the issue of patentability and official approval by European Authorities [35]. In the following section, we will discuss regulatory, production and clinical trial challenges to the establishment of phage therapy as a regulatory approved therapy.

### 3.2. Regulatory Challenges

The European Medicines Agency (EMA) held a workshop in 2015 together with relevant stakeholders including academia, industry, policy makers and patient organizations to identify possibilities for the development of bacteriophage-based therapies against bacterial infections [94]. Approximately 60 experts discussed practical and regulatory issues related to phage licensing pathways as opposed to conventional medicine, for example, whether or not the EU Directive 2001/83/EC (relating to medicinal products for human use) might be applicable for phages [84,94,95]. As EMA has not licensed any phage products so far, it is not clear which pathway to approval is most promising. Moreover, modification or updating of existing phage cocktails with new phages, necessities with regard to developing phage resistances or changing pathogens, are not yet covered by existing regulations [84]. Hence, currently new time and cost intensive re-production and re-approval under GMP conditions would be required [84]. As phage biology (and bacterial co-evolution) implies the necessity for fast turnover of specific phages in clinical use, the process of development and approval needs to be shortened, which could be achieved by approval of production processes rather than specific phage products. Additionally, it must be clarified whether each phage of a cocktail or the complete cocktail is considered a medicinal product and needs regulatory approval.

### 3.3. Production Challenges

For a broad medical application, phages have to be produced in large scale by pharmaceutically licensed facilities. Consequently, there will be a commercial interest in the optimization of processes and the reduction of costs, which is indeed not trivial as non-linear dynamics of phages and host bacteria have to be considered [96]. In order to scale up phage manufacturing Krysiak-Baltyn et al. [96] proposed a computational model appropriate for modelling phage production, including varying infection parameters. This model might be suitable to cut costs or to improve productivity [96]. With the increasing interest in phage therapy, the issue of intellectual property (IP) protection comes to the fore [97]. To date the options for IP protection of naturally occurring phages are limited because there are abundant resources to isolate phages from the environment [36]. Therefore, other possibilities should be considered. For example, there are several options to implement IP protection in the manufacturing process, for example, every new phage, new preparation (consisting of approved single phages) or the production method, in order to increase attractiveness of the field to economic stakeholders [39,98,99].

### 3.4. Clincial Trial Challenges and Ongoing Projects

Recently, results of the first European randomized, controlled phase 1/2 trial aimed at evaluating the efficacy and tolerability of a topical applied phage cocktail (PP1131) against *P. aeruginosa* in burn wounds (PhagoBurn, www.phagoburn.eu) have been published [79]. The authors reported that patients treated with the phage cocktail showed slower decrease of bacterial burden in the burn wounds compared to patients receiving standard therapy (1% sulfadiazine silver emulsion cream). This finding must be interpreted with caution, as the study had several limitations: The patient cohort was relatively small (standard therapy *n* = 13, PP1131 *n* = 12) and inhomogeneous, as patients treated with phages were older, were burned to a lesser extent and showed higher bacterial burden at therapy start compared to those treated with standard therapy [79]. Importantly, there have been some stability issues of the phage cocktail used, as the authors reported a decrease in plaque forming units (pfu) during the study, which was associated with the application of a lower than intended dose of active phages. Future studies should address stability and shelf-life of each phage product including phage cocktails. The relatively large cocktail of 12 phages resulted in double the expected production time and thus reduced time to recruit patients [79]. Although the study terminated prematurely due to insufficient efficacy, it was the first trial using a cocktail of phages purified according to GMP standards and approved by national health regulators [79].

The German Phage4Cure (http://phage4cure.de/) consortium aims to address the safety, tolerability and efficacy of a purified inhaled bacteriophage cocktail against chronic airway infection with *P. aeruginosa* [100]. The goal of the four project partners (Fraunhofer Institute for Toxicology and Experimental Medicine, ITEM; the Leibniz Institute DSMZ-German Collection of Microorganisms and Cell Cultures GmbH; Charité–Universitätsmedizin Berlin; and Charité Research Organisation GmbH, CRO) is to pave the way for clinical applications of bacteriophages in Germany and Western Europe by applying GMP standards in the entire production chain of the phage product and by getting approval for phage therapy from regulatory authorities [100]. The project gained governmental financial support (funded by the German Federal Ministry of Education and Research) and regulatory authorities, namely BfArM (Federal Institute for Drugs and Medical Devices), are closely involved [100]. The Phage4Cure team is focusing on bacteriophages targeting *P. aeruginosa*, which is characterized by high abundance, rapid growth, distinct ability to form biofilms and a highly flexible genome, aspects that contribute its wide distribution and difficulties in combatting this bacterium [101]. *P. aeruginosa* is intrinsically resistant to several classes of antibiotics and there is increasing evidence of strains resistant to antibiotics of last resort [101]. Immunocompromised patients and patients with pre-injured lungs, particularly those with cystic fibrosis (CF) are frequently colonized by bacteria with 80% of CF patients older than 18 years harbouring *P. aeruginosa* [102]. CF is a genetic disorder caused by a mutation in the cystic fibrosis transmembrane conductance regulator (CFTR) gene that affects the lungs, as well as the pancreas, liver, kidneys and intestine [103]. Non-cystic fibrosis (non-CF) bronchiectasis represents a chronic and heterogeneous airway disease with diverse aetiology. Like patients with CF, non-CF bronchiectasis patients are highly susceptible to pulmonary infections. Most patients with bronchiectasis are colonized with antibiotic resistant bacteria (e.g., *Acinetobacter, Pseudomonas, Burkholderia*), with *P. aeruginosa* infections being associated with poor prognosis [104,105,106,107,108]. As patients with bacterial colonization but not infection are clinically relatively stable, effectiveness and tolerability of inhaled phage therapy may well be tested. Consequently, *P. aeruginosa* colonization in CF and non-CF bronchiectasis patients was chosen by the consortium Phage4Cure as therapeutic target to pave the way to a clinical trial with phages produced under GMP conditions and, ultimately, to regulatory approval. On the basis of their expertise, each partner will work on different aspects of the project, ranging from phage selection and characterization (DSMZ; as discussed by Korf et al. in this issue of the journal) to drug production and stability testing (ITEM). Following phage production and thorough preclinical evaluation (Charité and ITEM), clinical phase 1 and 2 trials are planned to be performed at Charité. If successful, the project may lead to first-time establishment of phages as approved inhaled therapy for CF and non-CF bronchiectasis patients in Germany and may possibly provide a GMP-compliant phage purification platform process as a blueprint for phage therapy development with respect to other indications [6,100]. Further projects related to the implementation of phage therapy in Western Europe include PhagoMed and PhagoFlow. The biotech company PhagoMed Biopharma GmbH (https://www.phagomed.com/), based in Vienna, focuses on the development of phage-based therapies for bacterial infections [109]. Supported by grants and private investments, PhagoMed evaluates, inter alia, the treatment of infected prostheses with phages. PhagoFlow aims at testing a magistral prescription of phages in patients with wounds infected by MDR bacteria and is being carried out at the military hospital Berlin, together with DSMZ and Fraunhofer ITEM [110]. Magistral preparation in the EU is defined as “any medicinal product prepared in a pharmacy in accordance with a medical prescription for an individual patient” (Article 3 of Directive 2001/83 and Article 6 quarter, § 3 of the Law of 25 March 1964) [111] and is therefore a practical way to produce treatments adjusted to the special needs of an individual without being dependent on commercial manufacturing [111]. In Belgium, the magistral preparation is already used for phages [111] and could provide a solution for individual patients but cannot cover the requirements of a larger patient cohort. GMP and GCP guidelines also apply for magistral applications. To promote a European solution to conquer the regulatory challenges of personalized and phage based medicinal products, the idea of a “biological master file”, a concept already existing for chemical drugs but not for biologically active substances, was suggested by Fauconnier [112]. In summary, the main prerequisites for the establishment of a bacteriophage-based therapy are specific regulations for phage-based pharmaceuticals, increased clinical trial evidence and an infrastructure for efficient and rapid phage provision [6,36,113].

After decades of sleeping like Rip van Winkle, phage therapy is currently awakening in Western Europe due to the noise made by antimicrobial resistance, causing relevant research activity in the field. New valuable data addressing current concerns regarding clinical use of phages can be expected. However, whether phages will be approved by regulatory authorities and get market access is currently unpredictable.
